# Evaluation of binding activities of a putative lipoprotein LIC_13355 of *Leptospira* spp.

**DOI:** 10.1002/2211-5463.13942

**Published:** 2024-12-12

**Authors:** Igor R. M. Silva, Maria B. Takahashi, Aline F. Teixeira, Ana L. T. O. Nascimento

**Affiliations:** ^1^ Laboratório de Desenvolvimento de Vacinas Instituto Butantan São Paulo Brazil; ^2^ Programa de Pós‐Graduação Interunidades em Biotecnologia, Instituto de Ciências Biomédicas Universidade de São Paulo Brazil

**Keywords:** *Leptospira*, leptospirosis, pathogenesis, recombinant protein

## Abstract

Pathogenic *Leptospira* is the etiological cause of the zoonotic life‐threatening infection called leptospirosis. The disease is spread worldwide with higher risk in tropical regions. Although leptospirosis represents a burden to the health of humans and animals, the pathogenic mechanisms of *Leptospira* infection are yet to be clarified. Leptospirosis infection is multifactorial, involving functionally redundant proteins with the capability to invade, disseminate, and escape the host's immune response. In this work, we describe a putative lipoprotein encoded by the gene LIC_13355, genome annotated as hypothetical of unknown function. The coding sequence is conserved among pathogenic *Leptospira* spp. with high percentage of coverage and identity. The recombinant protein, rLIC_13355, was expressed in *Escherichia coli* host system in its insoluble form. The circular dichroism spectrum of the refolded protein showed it containing a mixture of secondary structures. rLIC_13355 interacts with extracellular matrix (ECM) component laminin and binds plasminogen (PLG), generating plasmin (PLA), thus possibly participating during the adhesion and dissemination processes. The rLIC_13355 has the ability to interact with Ea.hy926 and HMEC‐1 endothelial cells either in monolayer or suspension. The binding of rLIC_13355 with monolayer cells is dose‐dependent on protein concentration. Taken together, our data suggest that this is presumably an adhesion lipoprotein that may play diverse roles in host–*Leptospira* interactions by mediating the interaction with host components and with endothelial cell.

## Graphical abstract

Pathogenic *Leptospira* causes leptospirosis, a severe zoonotic infection. Despite its impact, the infection mechanisms are unclear and likely multifactorial. We identify a putative lipoprotein, LIC_13355, conserved among pathogenic *Leptospira* spp. The recombinant protein rLIC_13355 interacts with extracellular matrix laminin and binds plasminogen, suggesting roles in adhesion and dissemination. It also binds endothelial cells, indicating potential involvement in host interactions.

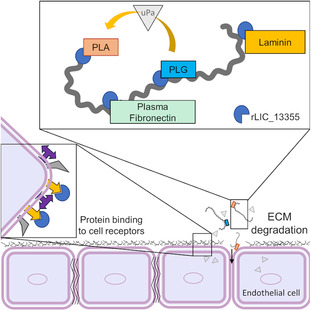

**Abbreviations**
BSA: bovine serum albumin
CD: circular dichroism
CS: calfskin
ECM: extracellular matrix
EMJH: Ellinghausen–McCullough–Johnson–Harris
FBS: fetal bovine serum
HRP: horseradish peroxidase
IPTG: isopropyl β‐d‐1‐thiogalactopyranoside
mAbs: monoclonal antibodies
NHS: normal human serum
OMP: outer membrane protein
OPD: o‐phenylenediamine
PBS: phosphate‐buffered saline
PBS‐T: phosphate‐buffered saline + 0.05% Tween 20
PDB: Protein Data Bank
PLA: plasmin
PLG: plasminogen
PMSF: phenylmethylsulfonyl fluoride
RT: rat tail
SD: standard deviation
SDS: sodium dodecyl sulfate
SDS/PAGE: SDS polyacrylamide gel electrophoresis
uPA: urokinase‐type plasminogen activator

Leptospirosis is a widespread zoonosis of global concern caused by pathogenic bacteria of the genus *Leptospira*. It is considered a re‐emerging disease that affects humans and animals. In urban settings, rodents are asymptomatic reservoirs of leptospires, shedding them live in the urine, contaminating the environment [1, 2]. The incidence of human leptospirosis is low in developed countries, mostly associated with recreational or occupational activities [3, 4], while in developing countries, the high incidence is related to poor sanitation conditions. The leptospires penetrate the host via intact, sodden, damaged skin, or mucosa. Infection produces a wide spectrum of clinical flu‐like symptoms, such as fever, chills, headache, and myalgias, characterizing the onset of the disease. The disease may evolve to multi‐organ complications known as Weil's syndrome and leptospirosis pulmonary hemorrhage syndrome, reaching up to 15% and 70% mortality rates, respectively [5, 6]. Leptospirosis has an economic impact as the disease affects livestock and causes abortions, stillbirths, infertility, reduced milk production, and death [1].

The fundamental mechanism in the pathogenesis of leptospirosis, as in other diseases, is the capacity of the pathogens to propagate in the host at the beginning of infection [7]. In this sense, it is anticipated that surface‐associated proteins may promote various activities, including adhesion, antigenicity, and host cell stimulation [8]. Accordingly, pathogenic *Leptospira* must have the ability to adhere, to invade, and to evade the immune system to colonize diverse tissues and organs of the host. Investigating the strategies involved in these activities should contribute to our understanding of the infection process.

Several leptospiral genomes have been sequenced [9, 10, 11, 12, 13], and functional genomics, proteomics, and host–pathogen interactions are being actively investigated. However, to date, only a few virulence factors have been demonstrated, and the basis of pathophysiological leptospirosis symptoms and the gravity of the disease are yet to be elucidated [9, 10, 11, 12, 14, 15, 16]. The identification of proteins that could participate in those processes can be critical for the development of vaccines, treatments, and diagnostic methods.

The genome sequences of *Leptospira interrogans* serovar Copenhageni strain Fiocruz L1‐130 [17] revealed 184 putative and chemically identified lipoproteins [18]. These findings were followed by studies, in which adhesion to host components and cellular receptors were attributed to lipoproteins [19, 20, 21, 22, 23, 24, 25]. The possible role of lipoproteins in spirochaetal pathogenesis has been well discussed by Haake *et al*. [26]. LipL21 and the previously known outer membrane proteins, LipL32 and LipL41, were shown to have putative role in leptospiral infection [26, 27, 28, 29], while LipL46 was expressed during leptospiral dissemination in the mammalian host [30]. Besides lipoproteins, several other proteins of *L. interrogans* have been reported to bind/generate plasminogen (PLG)/plasmin (PLA) [31, 32, 33], to bind components of complement system [34, 35], and to mammalian cell [36, 37], such as OmpL37, an elastin‐binding protein of pathogenic *Leptospira* [38]. These proteins could be involved in leptospiral pathogenesis.

In this study, we characterized and examined the binding properties of a putative lipoprotein of *Leptospira* spp., encoded by the gene LIC_13355, genome annotated as hypothetical, and predicted to be a lipoprotein. We evaluated the binding of the recombinant protein rLIC_13355 to host components and its adhesion to mammalian cells. As control, the proteins LipL46, LipL41, and Ompl37 were used. The results suggest that this is a multipurpose protein capable of participating in various steps of the infection process.

## Materials and methods

### Biological components

Collagen, laminin, plasma and cellular fibronectin, elastin, fibrinogen, human vitronectin, and the control protein BSA were purchased from Sigma‐Aldrich (St. Louis, MO, USA). Laminin‐1 and collagen type IV were derived from the basement membrane of Engelbreth‐Holm‐Swarm mouse sarcoma, while cellular fibronectin was derived from human foreskin fibroblasts and elastin from human aorta, and collagen type I was isolated from rat tail (RT) and calfskin (CS). Native plasminogen was purchased from EMD (San Diego, CA, USA).

### Bacterial strains and mammalian cell cultures

Pathogenicity‐attenuated *L. interrogans* serovars Canicola strain Hond Utrecht IV, Copenhageni strain M20 was cultured at 28 °C under aerobic conditions in liquid EMJH medium containing asparagine (0.015% w/v), sodium pyruvate (0.001% w/v), calcium chloride (0.001% w/v), magnesium chloride (0.001% w/v), peptone (0.03% w/v), and meat extract (0.02% w/v) [39]. *Escherichia coli* DH5α and *E. coli* BL21(DE3) Star pLys [40] were used as cloning and recombinant protein expression hosts, respectively. Leptospiral DNA extraction was performed as described previously [41]. Cell lines were purchased from ATCC (Manassas, VA, USA). Endothelial cell lines HMEC‐1 (human dermal microvascular, ATCC CRL‐3243) and Ea.hy926 (human endothelial macrovascular somatic cell hybrid, ATCC CRL‐2922) were used. Cell line HMEC‐1 was grown in MCDB 131 medium (M8537; Sigma‐Aldrich) supplemented with 20% fetal bovine serum (FBS; Bionutrientes, São Paulo, SP, Brazil), 10 ng·mL^−1^ epidermal growth factor (E9644; Sigma‐Aldrich), 1 μg·mL^−1^ hydrocortisone (H0888; Sigma‐Aldrich), and 10 mm glutamine (ATCC), while Ea.hy926 was grown in Dulbecco's modified Eagle medium (DMEM, D7777; Sigma‐Aldrich) supplemented with 10% FBS and 2 mm glutamine. Cells were grown at 37 °C under 5% CO_2_ atmosphere and tested for mycoplasma contamination before the assays.

### *In silico* analysis of the LIC_13355 coding sequence

The coding sequence of *L. interrogans* serovar Copenhageni str. Fiocruz L1‐130 for LIC_13355 was retrieved from the GenBank database (https://www.ncbi.nlm.nih.gov/genbank/) [42], which was analyzed for cellular localization via cello (http://cello.life.nctu.edu.tw/) [43], psortb (https://www.psort.org/) [44], and sosuigramn (https://harrier.nagahama‐i‐bio.ac.jp/sosui//sosuigramn/sosuigramn_submit.html) [45]. SignalP (SignalP‐6.0 webserver; https://services.healthtech.dtu.dk/services/SignalP‐6.0/) [46] webserver was used to predict signal sequence and the presence of signal peptidase sites. Potential domains were analyzed using interpro scan (https://www.ebi.ac.uk/interpro/search/sequence/) [47, 48], smart (http://smart.embl‐heidelberg.de/) [49, 50], and psipred (http://bioinf.cs.ucl.ac.uk/) [51] programs. clustal omega [52] was used to compare the presence and conservation of coding sequence against pathogenic strains of *Leptospira* and against intermediate and saprophytic strains (https://www.ebi.ac.uk/jdispatcher/msa/clustalo). Tertiary structure was modeled using collabfold2 software, based on alphafold2 prediction algorithm (https://colab.research.google.com/github/sokrypton/ColabFold/blob/main/AlphaFold2.ipynb) [53, 54, 55].

### Cloning of LIC_13355, expression, and purification

The LIC_13355 sequence was amplified from *L. interrogans* M20 genomic DNA using a set of primers designed as follows: forward 5′‐ATCGGGATCCAAAGAAAATACCTCCGAC‐3′ (*Bam*HI) and reverse 5′‐ATCGCCATGGTTAATTCACCTGTTTGGC‐3′ (*Nco*I), with restriction sites added for cloning in pAE vector [56]. Primers were designed considering the removal of the signal peptide sequence. Amplicons were purified with Illustra GFx PCR DNA and Gel Band Purification kit (GE Healthcare, Chicago, IL, USA) and then cloned into pAE vector cloning site. DNA insert presence was confirmed by sequencing of recombinant plasmid on an automated ABI sequencer (PE Applied Biosystems, Foster City, CA, USA) [57] with the T7 (5′‐TAATACGACTCACTATAGGG‐3′) and pAER (5′‐CAGCAGCCAACTCAGTTCCT‐3′) primers. The cloned sequence has 100% identity with the corresponding in *L. interrogans* serovar Copenhageni, strain Fiocruz L1 130. BL21 Star™ (DE3) pLysS cells were transformed with the recombinant plasmid in Lysogeny broth (LB; 0.5% yeast extract, 1% tryptone, 0.5% NaCl) supplemented with antibiotics (50 μg·mL^−1^ ampicillin and 34 μg·mL^−1^ chloramphenicol) and grown overnight at 18 °C under agitation. From the saturated culture, 5 mL was taken to inoculate 300 mL of fresh medium, maintained under agitation at 18 °C. Upon reaching the mid‐log phase, determined by the culture's optical density at 600 nm (OD_600_; around 0.6), the recombinant protein was induced with 0.01 mm isopropyl β‐d‐1‐thiogalactopyranoside (IPTG) and kept under agitation at 18 °C for 16 h. The cells were collected by centrifugation (8000 ***g***, 10 min), resuspended in 30 mL of lysis buffer (10 mm Tris/HCl pH 8.0, 150 mm NaCl, 1× Triton X‐100, 20 mm phenylmethylsulphonyl fluoride (PMSF), and 0.2 mg·mL^−1^ lysozyme), and lysed by sonication in Sonifier 250 sonicator (Branson, Danbury, CT, USA). The fractions were separated by centrifugation (8000 ***g***, 10 min at 4 °C). The soluble supernatant was then separated, and the insoluble fraction was solubilized in 30 mL of denaturation buffer (10 mm Tris/HCl pH 8.0, 150 mm NaCl, and 8 m urea). For purification of the rLIC_13355 protein, the insoluble fraction of the culture was loaded onto a Chelating Sepharose chromatography column (GE Healthcare) with Ni^2+^. The protein bound to the column was washed with 10 mm Tris/HCl pH 7.4 buffer with decreasing urea concentration (8, 6, 4, 2 and 0 m) for refolding and purified by successive column washing with 10 mm Tris/HCl pH 7.4 buffers with increasing imidazole concentration (40, 60, 100, and 500 mm). Fractions were collected and analyzed by SDS/PAGE. The purified protein fractions were dialyzed against 10 mm Tris/HCl, 200 mm NaCl, pH 7.4 buffer, and concentration determined using the Bradford kit.

### Circular dichroism (CD) spectroscopy

Samples of rLIC_13355 were dialyzed in phosphate buffer (10 mm, pH 7.4) and submitted for analysis on a Jasco J‐810 spectropolarimeter (Japan Spectroscopic, Tokyo, Japan). Ten measures were taken using a 1‐mm path length cuvette at intervals of 0.5 nm·s^−1^ ranging from 190 to 250 nm. The spectra expressed in terms of residual molar ellipticity were analyzed using the DichroWeb program (http://dichroweb.cryst.bbk.ac.uk/), which calculates the secondary structure content of the protein based on experimental ellipticity data [58], using the K2D reference dataset [59].

### Antiserum production in mice immunized with rLIC_13355

Female BALB/c mice weighing between 18 and 22 g were subcutaneously immunized with 10 μg of rLIC_13355 adsorbed in Al(OH)_3_ (12.5% v/v per dose), with two booster doses administered at approximately 15‐day intervals. The animals were bled via the retro‐orbital plexus before the first immunization and 15 days after each subsequent immunization. The collected blood was kept at room temperature for about 30 min, and the clot was centrifuged at 2000 ***g*** for 5 min. After centrifugation, the collected serum was stored at −20 °C. The sera were titrated by ELISA, with 0.5 μg per well of recombinant protein diluted in 50 μL of 1× PBS pH 7.4 adsorbed onto a 96‐well plate (Costar® High Binding; Corning Incorporated, Kennebunk, ME, USA) for 16 h. The plates were washed with PBS‐T and blocked with 200 μL of 10% (v/v) BSA in PBS for 2 h at 37 °C. The mouse sera, diluted 1 : 100 in PBS/BSA solution, were incubated with a solution of PBS/BSA + 10% (v/v) *E. coli* extract for 2 h at 37 °C. The plates were washed three times with PBS‐T, and serial dilutions of the anti‐protein serum were made from 1 : 100 to 1 : 204 800, incubating the plate at 37 °C for 1 h. The plate was washed six times with PBS‐T, and the interactions were revealed using mouse anti‐IgG antibody conjugated with peroxidase (1 : 5000 v/v; Sigma‐Aldrich). The reactions were developed using 100 μL of 1 mg·mL^−1^ o‐phenylenediamine (OPD; Sigma‐Aldrich) solution in 0.1 m sodium citrate + 0.2 m sodium phosphate (citrate–phosphate) at pH 5.0 and 1 μg·mL^−1^ H_2_O_2_, and the plate was kept in the dark for 10 min. Then, 50 μL of 4 N H_2_SO_4_ were added to stop the reaction, and absorbance at 492 nm was measured.

### Detection of LIC_13355 in leptospiral cell extracts

Detection of native protein was made using secreted and SDS‐soluble bacterial membrane proteins, as previously described [60]. Briefly, 100 mL of virulent low passage *L. interrogans* serovar Copenhageni strain Fiocruz L1‐130 culture was centrifuged, the medium discarded, and the pellet washed with 10 mL of PBS. Cells were resuspended in 10 mL PBS and incubated overnight at 30 °C for protein secretion. Leptospiral cells were pelleted, and the supernatant recovered and concentrated 10‐fold using 10 kDa cut‐off Amicon ultrafiltration spin column (Millipore, Burlington, VT, USA) and dialyzed against 10 mm ammonium bicarbonate. For SDS‐soluble membrane proteins, cells were lysed with three successive rounds of sonication for 30 s at 20 kHz. Whole cells were removed by centrifugation (3000 ***g***, 15 min), the supernatant separated and ultracentrifuged at 27 000 ***g*** for 20 min at 4 °C. The pellet was resuspended in PBS containing 2% SDS and stirred at 37 °C overnight. The SDS‐soluble fractions were collected after centrifugation at 17 000 ***g*** for 20 min at room temperature. The SDS extraction was repeated once at 37 °C for 4 h. SDS in the samples was removed using extract gel columns and SDS‐Out SDS precipitation kit (ThermoFisher Scientific, Waltham, MA, USA). Secreted and SDS‐soluble proteins were analyzed by western blotting, probed with polyclonal anti‐rLIC_13355 antibody (1 : 4000 v/v) for 2 h at room temperature, followed by detection with HRP‐conjugated anti‐mouse IgG (1 : 5000 v/v; R&D Systems, Minneapolis, MN, USA) for 2 h at room temperature.

### Binding assays of rLIC_13355 with ECM and plasma components

The reactivity assays of rLIC_13355 against host components, laminin, cellular fibronectin, collagen type I, collagen type IV, elastin, plasma fibronectin, plasminogen, vitronectin, fibrinogen, and the cellular receptor e‐cadherin, was performed by ELISA, following the protocol described by Atzingen *et al*. [19]. In this assay, 1 μg of purified host components was diluted in 100 μL of 1× PBS (pH 7.4) and then adsorbed onto treated 96‐well plates (Costar® High Binding; Corning Incorporated) at 4 °C for 16 h. BSA and fetuin were used as negative controls. The plate was washed three times with 1× PBS (pH 7.4) + 0.05% Tween 20 (PBS‐T) to remove unbound solution and contaminants. Subsequently, the wells were blocked with 200 μL of 1× PBS‐T (pH 7.4) supplemented with 1% BSA (PBS‐T/BSA) at 37 °C for 2 h, followed by three washes with PBS‐T. In each well, a defined amount of component or recombinant protein diluted in PBS‐T/BSA was added, and the plate was further incubated for 1 h at 37 °C. Afterward, a specific HRP‐conjugated anti‐His tag mAbs (diluted 1 : 10 000 v/v; Sigma‐Aldrich) was added and allowed to react for 1 h at 37 °C. The plate was then washed six times with PBS‐T. The reactions were revealed using 100 μL of 1 mg·mL^−1^ OPD (Sigma‐Aldrich) in citrate–phosphate buffer pH 5.0, along with 1 μg·mL^−1^ H_2_O_2_. The plate was kept in the dark for 10 min, and then, 50 μL of 4 N H_2_SO_4_ were added to stop the reactions. The absorbances of the plates were measured at 492 nm. Statistically relevant binding was evaluated by the two‐tailed Student *t*‐test (*P* < 0.05) against negative controls in graphpad prism v6.05 (Boston, MA, USA).

### Dose–response curve

Components that presented statistically significant interaction, related to control, were tested to determine the dose–response curve. In ELISA plates, 1 μg of each component was diluted in PBS. The plates were washed three times, blocked with PBS‐T/BSA, and then, 1 μg of rLIC_13355 was added to the first well of each row. The protein was serially diluted in 1 : 1 proportion and incubated for 1 h at 37 °C. After three washes with PBS‐T, HRP‐conjugated anti‐His tag mAbs (1 : 10 000 v/v; Sigma‐Aldrich) was added to the wells, and the plate was incubated at 37 °C for 1 h. The plates were washed again with PBS‐T and revealed as described in the previous section. Curves were prepared and dissociation constant determined in graphpad prism v6.05, using the ‘non‐linear regression’ tool, considering saturation binding with ‘one site‐specific binding’.

### Evaluation of PLG conversion into PLA

In this assay, 1 μg of rLIC_13355 was adsorbed onto 96‐well plates (Costar® High Binding; Corning Incorporated). The recombinant protein LipL46 [61] and BSA were used as the positive and negative control, respectively. The plates were washed three times with PBS‐T and blocked with PBS/BSA for 2 h at 37 °C. After blocking, 1 μg of PLG was added to the wells, with a control included where no PLG was added, and the plate was incubated for 2 h at 37 °C. Following washing, combinations of 5 ng of uPA (urokinase‐type PLG activator) and 0.8 mm PLA chromogenic substrate (d‐val‐leu‐lys‐4‐nitroanilide; Sigma‐Aldrich) were added to the wells. The combinations included uPA + substrate, uPA only, and substrate only. The plate was incubated for 16 h at 37 °C and read at 405 nm. Statistical analysis was performed using graphpad prism v6.05, applying the two‐tailed Student *t*‐test against negative controls.

### Determination of rLIC_13355 binding to mammalian endothelial cells

To test the binding of rLIC_13355 to cells in monolayer, a 96‐well culture plate (Falcon 353916; Corning Incorporated) was seeded with 10^5^ cells per well in 100 μL of supplemented medium. For control, wells were coated with complete medium only. The plate was incubated for 16 h at 37 °C under 5% CO_2_ atmosphere. The plate was washed three times with PBS‐T and to each well was added 1 μg of recombinant protein in 100 μL of non‐supplemented medium with 1 μm aprotinin. The plate was incubated for 2 h at 37 °C under 5% CO_2_. Wells were washed with PBS‐T, and interactions were fixed by adding 100 μL of 2% paraformaldehyde in 1× PBS pH 7.4 to each well and incubating the plate at room temperature for 30 min. The plate was again washed, and 100 μL of 2% glycine in 1× PBS pH 7.4 were added to each well, followed by incubation of the plate at room temperature for 30 min. Wells were washed three times with PBS‐T and HRP‐conjugated 6×‐His tag mAbs (1 : 5000 v/v) added in 100 μL of 1× PBS pH 7.4, and the plate was incubated at 37 °C for 1 h. Plates were washed six times with PBS‐T and reactions revealed as described above. Binding of the rLIC_13355 protein was also assessed with cell suspension incubation. Cell culture monolayers in T75 flasks were trypsinized and resuspended with culture medium without supplementation (2 × 10^5^ cells per sample). Recombinant protein was added at three different concentrations containing 1 μm aprotinin to a final volume of 200 μL, and the cells were incubated at 37 °C under 5% CO_2_ for 2 h. Cells were centrifuged at 130 ***g*** for 5 min. Supernatant was removed and the pellet was resuspended in 200 μL of PBS. SDS sample buffer (50 μL) (250 mm Tris/HCl pH 6.8, 50% glycerol, 1% SDS, 1 mm β‐mercaptoethanol, 0.1% bromophenol blue) was added, and the samples were boiled at 96 °C for 10 min and analyzed by western blotting, along with an aliquot of purified protein used as positive control.

Protein was determined in the supernatant and pellet samples by western blot using polyclonal antibodies produced in mouse against recombinant protein, as described above. The purified recombinant protein was used as positive control.

### Ethics statement

All animal studies were approved by the Ethical Committee for Animal Research of the Instituto Butantan, Brazil, under protocol CEUAIB 2840200422. The Committee for Animal Research at Instituto Butantan adopts the guidelines of the Brazilian College of Animal Experimentation (COBEA).

## Results

### *In silico* analysis of the predicted coding sequence LIC_13355

The coding sequence LIC_13355 was analyzed by various programs; SignalP‐6.0 webserver predicts this coding sequence with a lipoprotein signal peptide (Sec/SPII), with a likelihood of 0.9013, and a cleavage site between amino acids 19 and 20 [62]. It suggests that the protein is processed, can be anchored in the bacterial membrane through their attached lipids. A signal peptide from amino acids 1 to 20 is the only conserved domain predicted by smart [49, 50]. The sosuigramn and cello programs predicted this coding sequence to be extracellular protein, while psortb predicted it as an outer membrane protein (OMP). Studies by Thoduvayil *et al*. [63], using Triton X‐114 fractionated subcellular proteome of *L. interrogans* reported significant amount of LIC_13355 in the outer membrane fraction. Extracellular proteome analysis revealed that a minor amount of LIC_13355 can be found on the leptospiral surface [64].

**Fig. 1:**
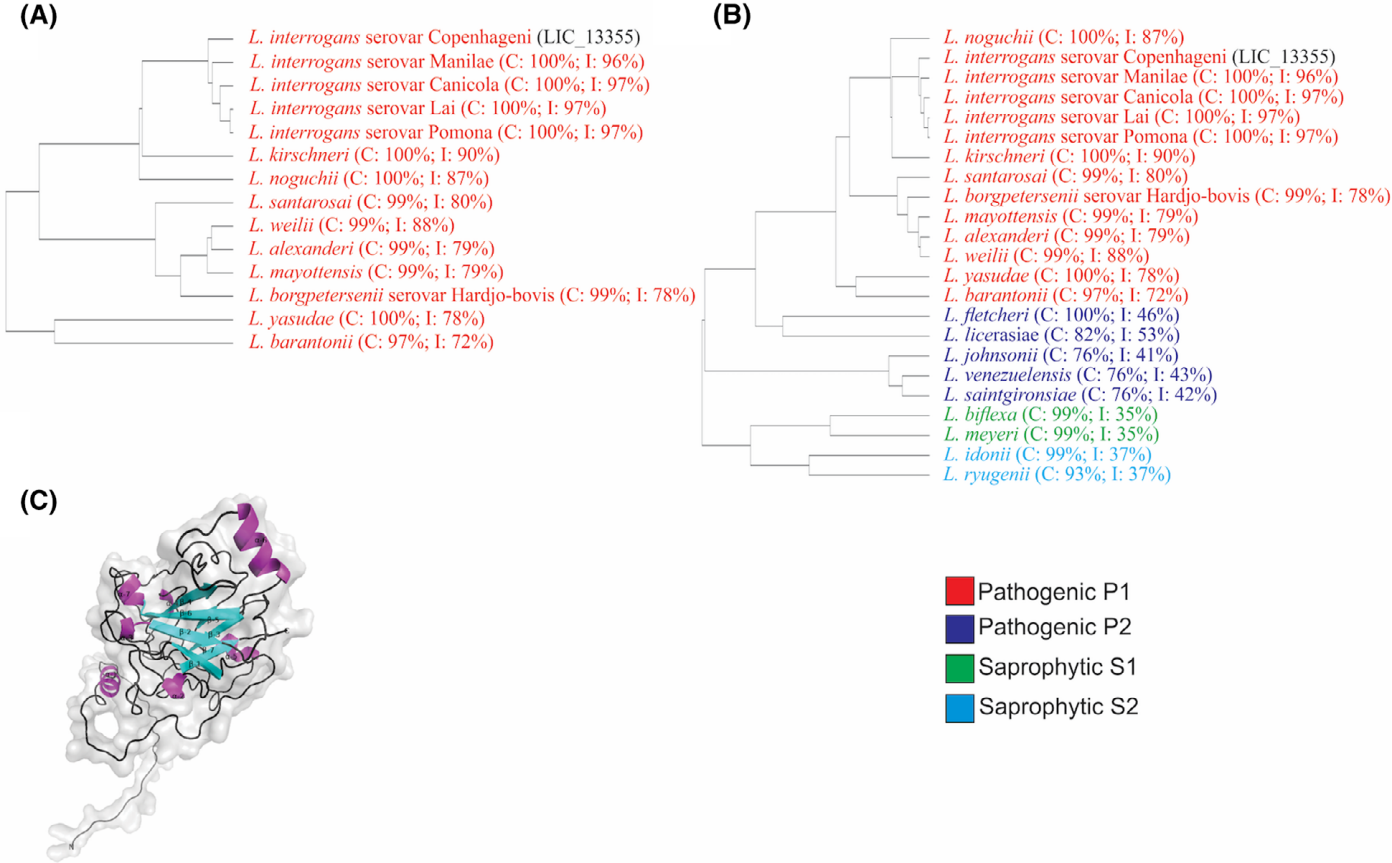
Bioinformatics and tertiary structure analysis. Conservation analysis of the coding sequence LIC_13355 of *Leptospira interrogans* serovar Copenhageni strain Fiocruz L1‐130 was implemented using blastp to retrieve the data and clustal omega program to sequence alignments. The search was performed against pathogenic strains of *Leptospira* (A) and against *Leptospira* spp. (B). Conservation with the clade P1 (in red), P2 in purple, S1 in green, and S2 in blue. C and I, stand for percentage of coverage and identity, respectively. (C) AlphaFold2 modeled tertiary structure: coil regions are colored in black, alpha helix shown in magenta, and beta sheets in cyan.

According to blastp analysis, the coding sequence LIC_13355 of *L. interrogans* serovar Copenhageni strain Fiocruz L1‐130 is a hypothetical, putative lipoprotein. clustal omega alignments of the sequences retrieved from blastp show that LIC_13355 is highly conserved among strains of pathogenic *Leptospira*, subclade P1, formerly described as the pathogen group (Fig. 1A), with a coverage ranging from 97% to 100% and identity from 72% to 97%. The alignments against *Leptospira* spp. (Fig. 1B) demonstrated the presence of LIC_13355 in pathogenic subclade P2, formerly described as the intermediate group, and saprophyte subclades S1, formerly described as the saprophyte group, and subclade S2, the new subclade that includes *L. idonii*, described in Vincent *et al*. [65], with a coverage ranging from 76% to 100% and identity from 35% to 53%. The data show higher identity of LIC_13355 among *Leptospira* spp. of subclade P1, suggesting a potential contribution to bacterial pathogenesis. AlphaFold2 modeled the tertiary structure of LIC_13355 (Fig. 1C) showing the coil regions which are colored in black, the alpha helix colored in magenta, and the beta sheets shown in cyan. This 3D matches two similar structures existing in the PDB, one of the *Plasmodium falciparum* PA28‐20S proteasome [66] and the other ChsB1 from *Mycobacterium tuberculosis* [67]. Both proteins seem to be involved in survival and persistence of the pathogens.

### Cloning, expression, and purification of rLIC_13355

**Fig. 2:**
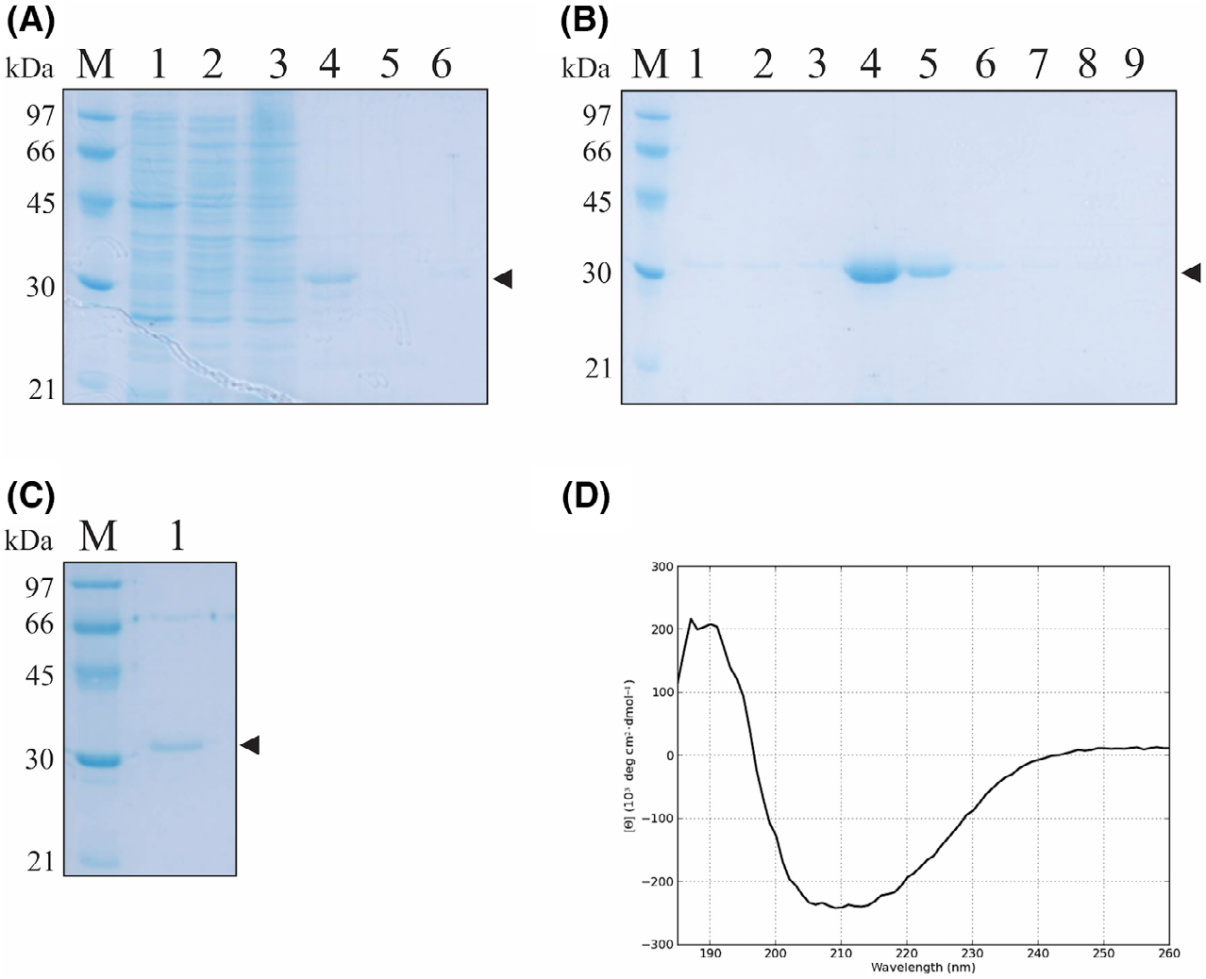
Expression, purification, and secondary structure of rLIC_13355. In (A–C), 12% SDS/PAGE analysis of rLIC_13355 expression, purification, and after dialysis. (A) M: Low molecular mass protein marker, lane 1: non‐induced *Escherichia coli* cell lysate, lane 2: cell lysate after induction with 0.01 mm IPTG, lane 3: soluble fraction, lane 4: insoluble fraction, lane 5: flowthrough from HisTrap column, lane 6: column wash with 60 mm imidazole. (B) Recombinant protein elution with 500 mm imidazole, lane numbers represent fractions collected. (C) Recombinant protein fraction after dialysis in 10 mm phosphate buffer pH 7.4. Arrows indicate the predicted molecular mass of rLIC_13355 protein. (D) Circular dichroism spectrum, representative of 10 experimental measures. Experimental spectra were recorded from 185 to 250 nm at 25 °C, using a 1 mm optical path cell with 0.5 nm intervals.

The coding sequence LIC_13355 was PCR‐amplified from the *L. interrogans* genome, without the signal peptide, and the generated amplicon was cloned into the pAE vector [56]. This vector adds 6×His to the N‐terminal of the recombinant protein, which facilitate its purification by metal‐chelating affinity chromatography. The pAE‐LIC_13355 was used to transform *E. coli* BL21 Star™ (DE3) pLysS and to express the recombinant protein. Studies on protein solubility showed that the rLIC_13355 is expressed in its insoluble form, as inclusion bodies, as depicted in the expression profile in Fig. 2A, lane 4. The recombinant protein was refolded in the column, with subsequent elution using increasing imidazole concentration, as shown in Fig. 2B. rLIC_13355 was dialyzed against phosphate buffer to remove imidazole and analyzed by 12% SDS/PAGE (Fig. 2C, lane 1). Deconvolution of circular dichroism data (Fig. 2D) indicated that the protein has a secondary structure component of 32% β‐sheet, 14% α‐helix, and 53% coil region. This is similar to that presented in modeled tertiary structure prediction built by AlphaFold2 and PSIPred analysis (see Fig. 1C).

### Immunogenic activity of rLIC_13355 in mice and western blotting

**Fig. 3:**
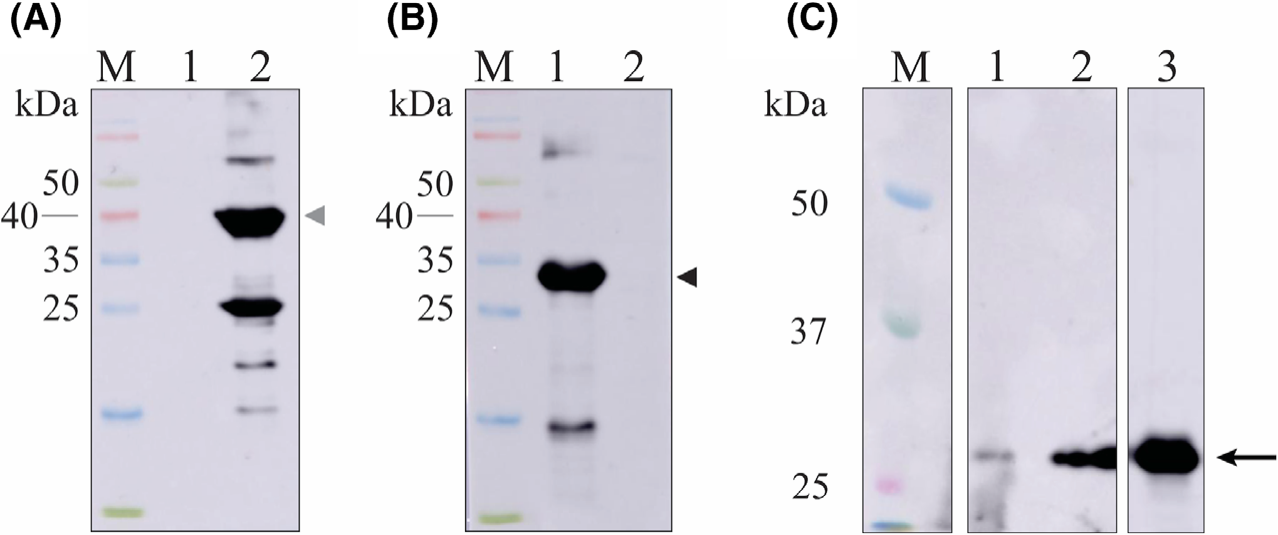
Western blotting of rLIC_13355 probed with antibodies. Protein (3 μg, each) rLIC_13355 and recombinant LipL41, used as a control, were run in a 12% SDS/PAGE and transferred into nitrocellulose membrane. In all membranes, M: molecular mass proteins, lane 1: rLIC_13355 and lane 2: recombinant LipL41. Membranes were probed with mouse polyclonal anti‐LipL41 (1 : 6000) (A) and with mouse anti‐rLIC_13355 (1 : 6000) (B). Protein bands were revealed with HRP‐conjugated anti‐mouse IgG (1 : 5000). Black arrows indicate the expected molecular mass for rLIC_13355 (28 kDa); gray arrow indicates the expected molecular mass for recombinant LipL41 (41 kDa). M: molecular mass protein marker. (C) LIC_13355 was recognized by mouse serum anti‐LIC_13355 in SDS bacterial cell extracts and secreted; M: protein molecular mass marker; 1: secreted; 2: SDS‐soluble protein; 3: Recombinant protein.

Purified rLIC_13355 was administered to BALB/c mice in three doses, with 14‐day interval. Serum titration revealed a high immunogenic activity, with a titer of 40 000 in the first immunization and 204 800 in second and third administration. The recombinant proteins rLIC_13355 and LipL41, used as control, were blotted onto nitrocellulose membrane and probed with anti‐LipL41 (1 : 6000) (Fig. 3A) and anti‐rLIC_13355 (1 : 6000) (Fig. 3B). Protein bands were revealed with HRP‐conjugated anti‐mouse IgG (1 : 5000). The expected band sizes of LipL41 and rLIC_13355 are indicated by the arrows, with the expected molecular weight of 41 and 28 kDa, respectively. The presence of the protein in *L. interrogans* L1 130 was attested by recognition of mouse serum anti‐LIC_13355 in bacterial cellular extracts either in the SDS fraction (major amount) or in extracellular (minor amount) (Fig. 3C).

### Interaction of rLIC_13355 with host components

**Fig. 4:**
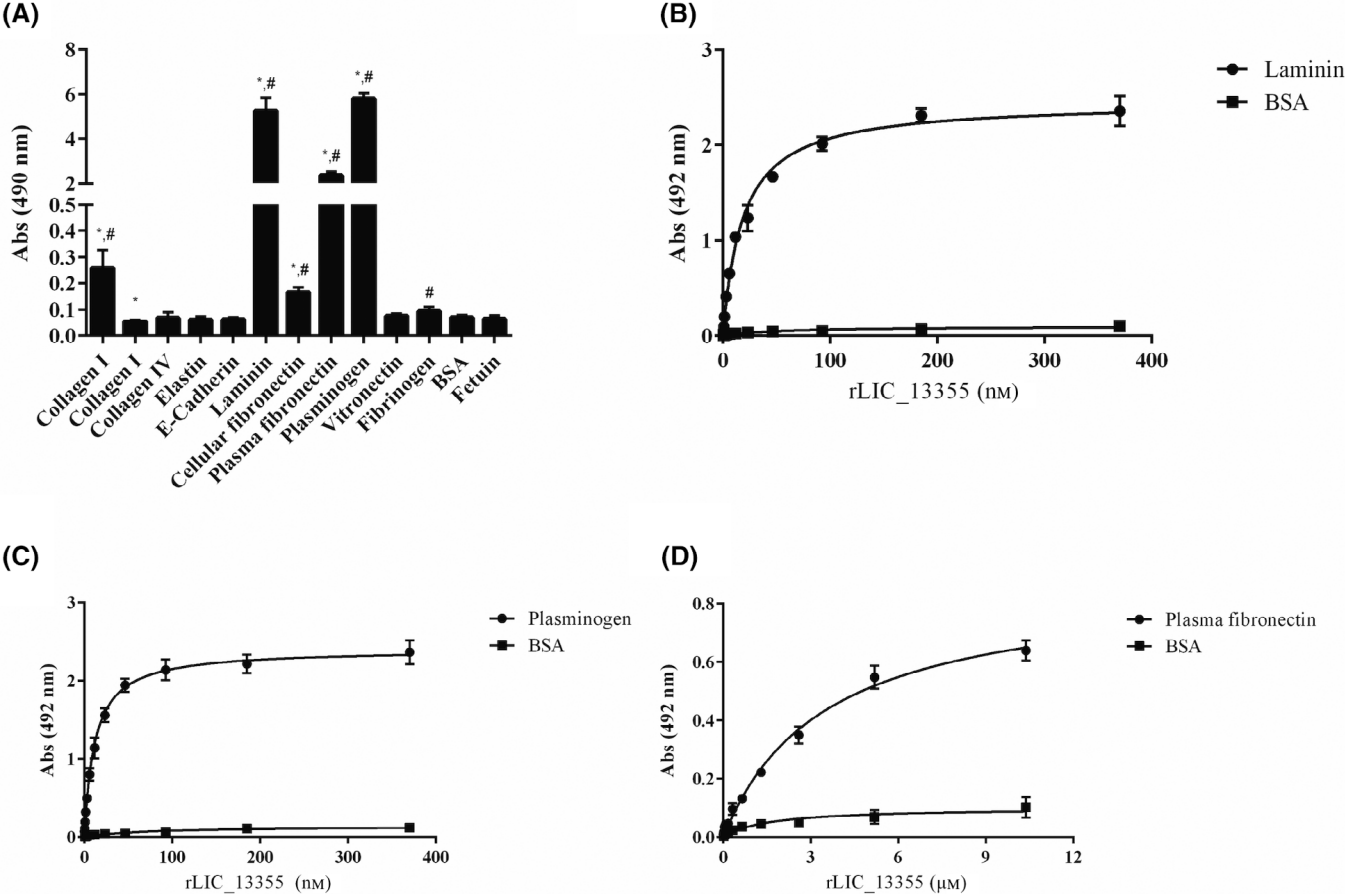
Protein binding screening to purified ECM and plasma components and rLIC_13355 dose–response plots. In (A), 1 μg of each component was immobilized in 96‐well plates, using BSA and fetuin as negative controls. Interaction with recombinant protein (1 μg) was conducted for 2 h. Bound protein was detected by HRP‐conjugated mAbs anti‐histidine (1 : 10 000 v/v). Component binding was compared to negative controls by two‐tailed *t*‐test (*against BSA and ^#^against fetuin, *P* < 0.05). Dose‐dependence of the binding in function of increasing rLIC_13355 concentration to laminin (B), to plasminogen (C), and to plasma fibronectin (D). BSA was used as negative control. Bars and dots represent the mean absorbance of three replicates. Each graph is representative of two independent experiments.

To investigate whether the recombinant protein interact with host constituents, ECM macromolecules, plasma components, and cellular receptor molecules were evaluated: laminin, cellular fibronectin, collagen type I, collagen type IV, elastin, e‐cadherin, elastin, plasma fibronectin, plasminogen, vitronectin, and fibrinogen. Fetuin and BSA were used as control proteins. Purified components were immobilized onto 96‐well plates, and recombinant protein attachment was probed with anti‐6× His mAbs, with the reaction assessed by ELISA. The results revealed binding of rLIC_13355 to plasma components, including PLG and plasma fibronectin, also to ECM components laminin, collagen type I, and cellular fibronectin (Fig. 4A). Binding to components that were statistically significant versus both controls, BSA and fetuin, were tested for their association by recombinant protein dose‐dependence assay. rLIC_13355 exhibited a high affinity for laminin (Fig. 4B) and PLG (Fig. 4C), with an estimated *K*_D_ of 18.49 and 11.78 nm, respectively. For plasma fibronectin, the estimated *K*_D_ was 3.49 μm, and no dose‐dependence was observed for the reaction with collagen type I and cellular fibronectin (data not shown). The data suggest that rLIC_13355 has a potential role in leptospiral colonization.

Table 1 displays the dissociation constant (*K*_D_) values observed for rLIC_13355 components and mammalian cell interactions.

**Table 1:** Dissociation constants (*K*_D_) of rLIC_13355 binding to purified host components and to monolayer endothelial human cells.

	Purified component		
	Laminin	Plasminogen	Plasma fibronectin
*K*_D_ (μm)	1.85 × 10^−2^ ± 0.121 × 10^−2^	1.18 × 10^−2^ ± 0.71 × 10^−3^	3.49 ± 0.31
	Endothelial cells		
	Ea.hy926	HMEC‐1	
*K*_D_ (nm)	25.91 ± 4.62	21.78 ± 7.74	

### PLG‐binding characterization

**Fig. 5:**
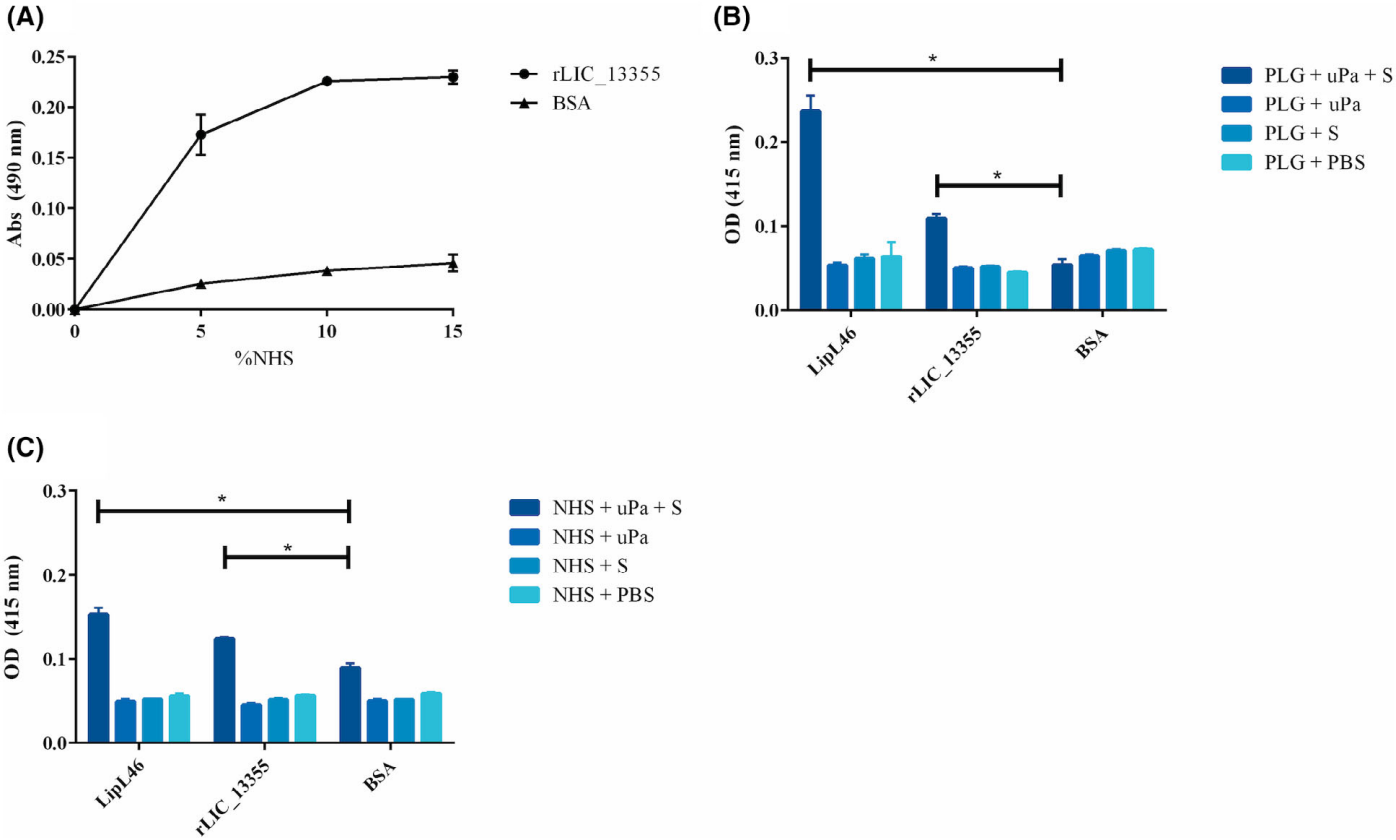
Characterization of PLG interaction with rLIC_13355. In (A), ninety‐six‐well plates were coated with 1 μg of recombinant protein or BSA, used as negative control. Wells were incubated with increasing concentrations of NHS (0–15%). Following incubation, plasminogen was detected using anti‐PLG antibody. The ligation was compared to BSA by two‐tailed *t*‐test (*P* < 0.05). In (B, C), bound PLG activation by urokinase PLG activator (uPA). ELISA plates were coated with recombinant LIC_13355, LipL46 or BSA, used as positive and negative control, respectively. Then, wells were incubated with PLG (1 μg) (B) or PLG‐captured from NHS (10% in PBS) (C), followed by the addition of uPA and PLA chromogenic substrate (d‐val‐leu‐lys‐4‐nitroanilide) for 16 h at 37 °C. PLG conversion to PLA were compared to negative control by two‐tailed *t*‐test (*P < 0.05). Bars and dots represent the mean absorbance of three replicates. Each graph is representative of two independent experiments.

It is reported that leptospires can bind to PLG, which is converted into PLA by host urokinase, conferring on the bacteria ECM‐proteolytic activity [68]. Furthermore, PLA generation reduces C3b and IgG deposition on the bacterial surface, facilitating immune evasion by decreasing opsonophagocytosis [33]. To evaluate whether rLIC_13355 contributes to these mechanisms, PLG uptake from normal human serum (NHS) was assessed by coating ELISA plates with rLIC_13355 and adding NHS at different concentrations. PLG binding was detected using specific anti‐PLG antibody (Fig. 5A). The protein was capable of capturing PLG from NHS in a dose‐dependent fashion. The high‐binding affinity of rLIC_13355 to PLG and its capacity to uptake it from NHS, prompted us to investigate if this zymogen could be converted into its active form, PLA, a broad‐spectrum serine protease, as previously shown with other leptospiral recombinant proteins [31, 69, 70]. For PLG conversion into its active form, PLA, a urokinase‐type PLG activator (uPa) was used, and conversion was indirectly detected with PLA chromogenic substrate (d‐val‐leu‐lys‐4‐nitroanilide). This experiment was performed using purified PLG (Fig. 5B) and PLG acquired from NHS (Fig. 5C). LipL46, a known PLG/PLA binding protein, was used as positive control [61]. In both cases, when the complete system was used (PLG, uPA, and PLA substrate), PLA was generated when bound to rLIC_13355, as observed with LipL46. The data suggest that LIC_13355 could mediate the generation of PLA on the leptospiral surface, contributing to the bacterial ECM degradation process.

### rLIC_13355 mammalian cell interaction

**Fig. 6:**
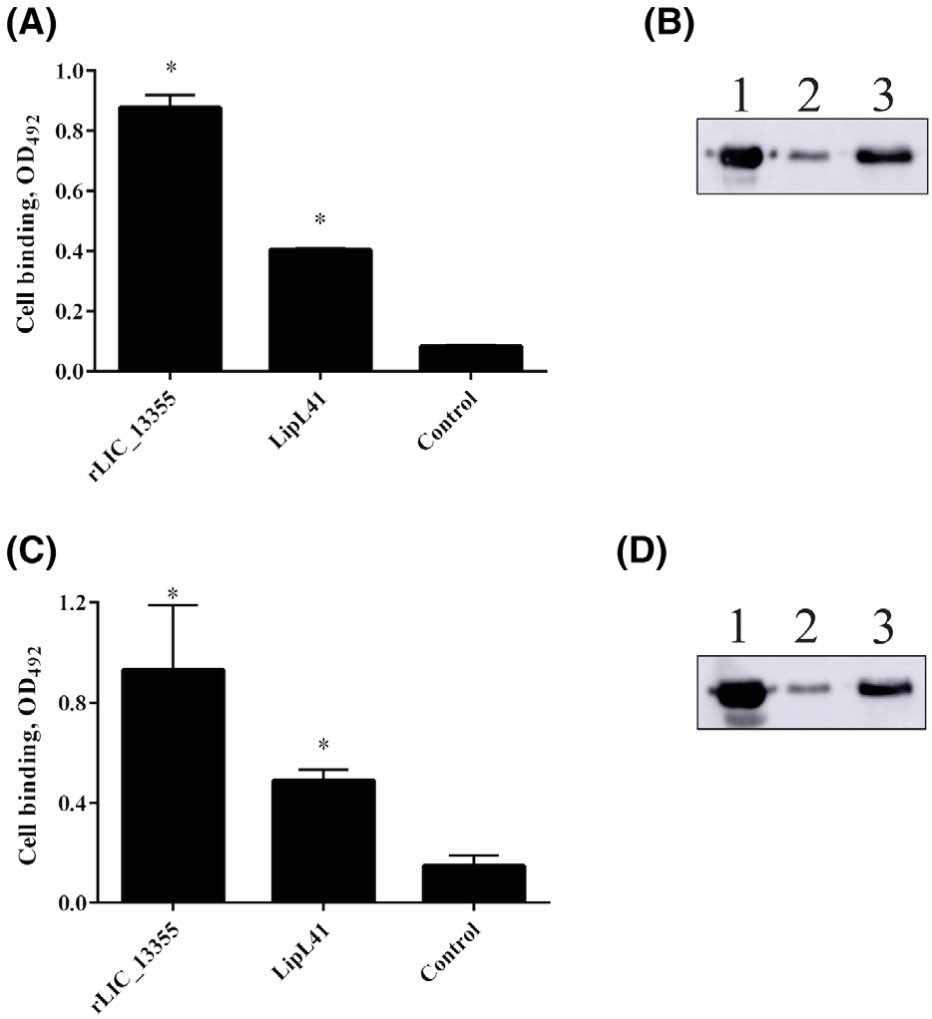
Interaction of rLIC_13355 with Ea.hy926 and HMEC‐1 endothelial cell lines. Binding of rLIC_13355 to mammalian cells were assessed with cells monolayers (immobilized assay) and detached cells (suspension assay). In (A), Ea.hy926 and (C), HMEC‐1 cell monolayers (left side): 10^5^ cells were seeded in 96‐well culture plates, with supplemented medium used as negative control. To wells, 1 μg of recombinant protein in 100 μL of non‐supplemented medium with 1 μm aprotinin were added, incubating the reaction for 2 h at 37 °C under 5% CO_2_. Reactions were fixed with 2% paraformaldehyde, then 2% glycine. Bound protein was revealed with HRP‐conjugated 6×‐His tag mAbs, and interaction was compared to negative control by two‐tailed *t*‐test (*P < 0.05). Bars represents the mean absorbance of three replicates. In (B), Ea.hy926 and (D), HMEC‐1 cell suspensions (right side): 10^5^ cells were incubated with 5 μg of recombinant protein in non‐supplemented medium with 1 μm aprotinin for 2 h at 37 °C. After trypsinization, cells were harvested by centrifugation, and fractions were analyzed by western blotting using HRP‐conjugated 6×‐His tag mAbs (1 : 10 000). Lanes 1: purified rLIC_13355, 2: cell pellet, 3: supernatant. Images are representative of two independent experiments.

**Fig. 7:**
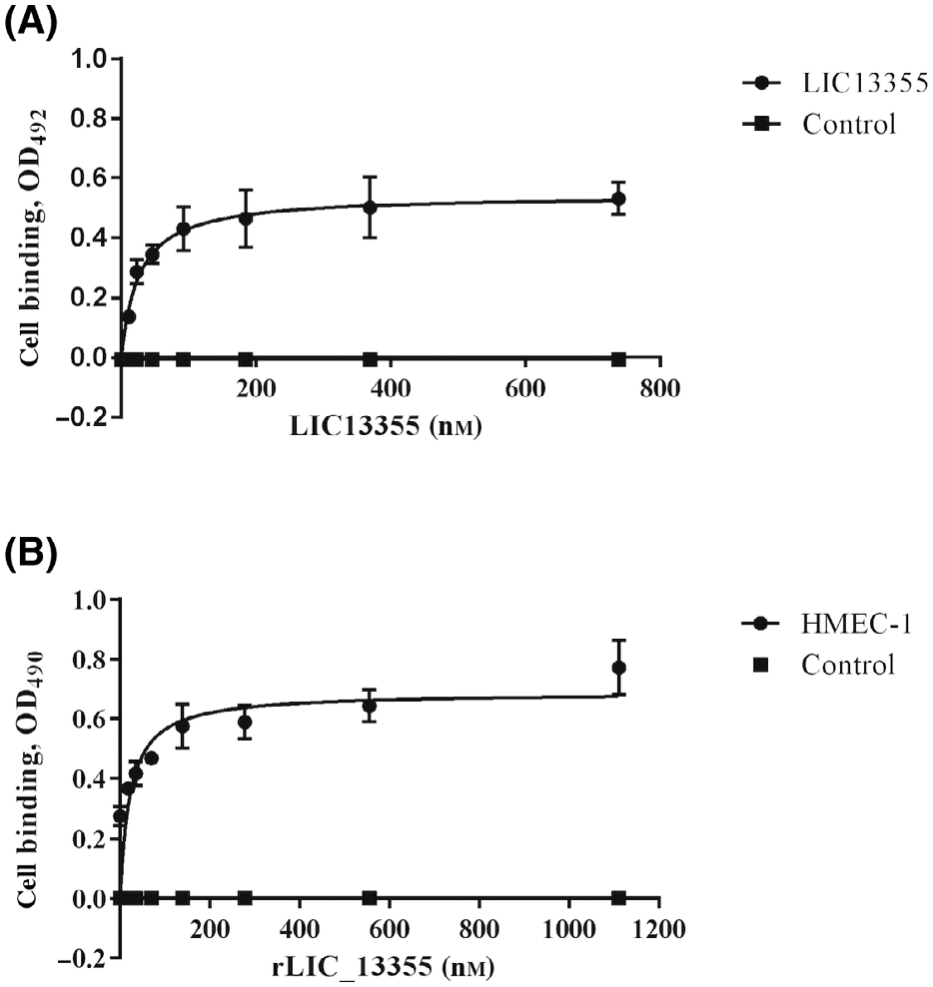
Binding of rLIC_13355 to Ea.hy926 and HMEC‐1 endothelial cell lines is dependent on protein concentration. Ninety‐six‐well plate were seeded with 10^5^ cell per well at 37 °C under 5% CO_2_ for 24 h. Supplemented medium was used ss negative control. Increasing concentration of recombinant protein was added, and the plate was incubated for 2 h at 37 °C under 5% CO_2_. Reactions were fixed by the addition of 2% paraformaldehyde, then 2% glycine. Binding was revealed by HRP‐conjugated 6×‐His tag mAbs (1 : 10 000). Curves were fitted and *K*_D_ calculated using graphpad prism software. Bar points represent the mean absorbance at 492 nm ± SD of three replicates; each graph is representative of two independent experiments.

It was reported that *Leptospira* strains, virulent, culture‐attenuated, and saprophytic, and the outer membrane proteins OmpL37, OmpL1, LipL21, LipL41, and LipL46 are able to adhere to different mammalian cell lines, including endothelial [36]. We decided to investigate whether rLIC_13355 could mediate this interaction with Ea.hy926 and HMEC‐1 endothelial cells. We evaluated attachment in cell monolayers and in cell suspensions by western blotting. We performed these assays using both conditions, aiming to determine whether binding is mainly to the ECM or to mammalian cell surface receptors. This is because when cells are harvested with trypsin, it detaches the cells from ECM, degrading components that are involved in cell adhesion [71]. In the case of cell monolayers, LipL41 was used as positive control. In both experimental conditions, rLIC_13355 was capable of binding to Ea.hy926 and HMEC‐1 endothelial cells in monolayers or suspension (Fig. 6). Binding of rLIC_13355 to monolayer was more effective with Ea.hy926 than with LipL41 (Fig. 6A); when binding was assessed with cell suspensions, part of the protein was found adhered to the cells (Fig. 6B, lane 2), while the majority was detected in the supernatant (Fig. 6B, lane 3). Similar results were found when the protein was tested with HMEC‐1 endothelial cells; the recombinant protein interacted with monolayer cells more effectively than with LipL41 (Fig. 6C); likewise, part of rLIC_13355 was detected in the cell pellet (Fig. 6D, lane 2), whereas the majority was found in the supernatant of HMEC‐1 cells (Fig. 6D, lane 3). The interactions between recombinant protein and endothelial cells were found to occur in a dose–response manner. Dose‐dependent binding was observed for the mammalian cells and rLIC_13355, reaching saturation at ~ 200 nm recombinant protein with both Ea.hy926 (Fig. 7A) and HMEC‐1 (Fig. 7B) endothelial cells. Thus, the coding sequence LIC_13355 has the ability to mediate the binding of *Leptospira* to mammalian cells.

## Discussion

LIC_13355 coding sequence is genome annotated as a hypothetical, putative lipoprotein of *L. interrogans*. Bioinformatics analysis shows that LIC_13355 possesses signal peptidase II sequence, a condition for the protein to be lipidated. Spirochetal lipoproteins have been associated with pathogenesis, and numerous of them have been identified in *L. interrogans* [26]. Some of them were confirmed experimentally as lipoproteins, such as LipL32 [26]; LipL21 [27], LipL41 [29], and LipL46 [30]. The LIC_13355 coding sequence is predicted to be extracellular or outer membrane protein and was identified in the outer membrane fraction by subcellular proteome and found with small amounts in extracellular proteome of *L. interrogans* [63, 64]. The coding sequence was shown to be well conserved with higher coverage and identity in subclade P1 of pathogenic *Leptospira* spp. When compared the modeled tertiary structure of LIC_13355 among the proteins with similar conformations structure existing in the PDB, the retrieved proteins with similar conformations were one of the *P. falciparum* PA28‐20S proteasomes, with implications for proteostasis in this organism [66], and the other ChsB1 from *M. tuberculosis* [67]. The first seems to be related to the sensitivity of malaria parasites to antimalarial drugs while the latter is involved with oxidation of cholesterol from the host that *M. tuberculosis* utilizes for survival, persistence, and virulence. Based on the tertiary structure similarity, we tentatively suggest the participation of LIC_13355 in leptospiral virulence.

LIC_13355 was cloned and expressed in *E. coli* BL21 Star™ (DE3) pLysS as 30 kDa full‐length recombinant protein, without the signal peptide, in its insoluble form. The purified protein exhibited a single major band in SDS/PAGE, suitable for further studies. The structural integrity of the purified protein after refolding was assessed by CD spectroscopy, which revealed a mixed population of β‐sheets, α‐helices, and coil regions. These regions are similar to the ones predicted in modeled tertiary structure (see Fig. 1C). The expression of LIC_13355 in *L. interrogans* L1 130 was confirmed by recognition with mouse serum anti‐LIC_13555 as surface and extracellular protein. The data are in agreement with proteomics studies previously reported [63, 64].

Putative lipoproteins, when produced as recombinant proteins, such as Lsa23, Lsa30, rLIC_10744, rLIC_12587, Lsa33, Lsa25, have been shown to bind ECM components, such as collagen IV, cellular fibronectin, laminin, and/or plasma components, for example, plasminogen and fibrinogen [20, 21, 25]. The predicted LIC_10507, LIC_10508, and LIC_10509 are located at the surface of pathogenic leptospires, expressed during infection of guinea pigs experimentally infected; these proteins induced the upregulation of ICAM‐1 and E‐selectin adhesion molecules in human umbilical vein endothelial cells (HUVECS) [72]. rLIC_10508 was reactive with leptospirosis human serum, which suggest its expression during human infection [37, 73]. Furthermore, studies by phage display screening showed that rLIC_10508 interacted with endothelial and epithelial cells *in vitro* [37]. Nonetheless, there is no sequence or structural relationship between LIC_10507, LIC_10508, and LIC_10509 proteins and LIC_13355. The interactions of LIC_13355 were dose–response and saturable, with high association constant (see Table 1). When bound to PLG, PLA was generated, either from the isolated PLG component or from NHS. This suggests that rLIC_13355 can contribute to PLA generation on the leptospiral surface, helping the bacteria in the degradation of ECM and evasion from the host immune system [33, 68].

The interaction of pathogenic *Leptospira* spp. and recombinant proteins with mammalian endothelial cells has been reported [36, 37, 74]. rLIC_13355 attachment to Ea.hy926 and HMEC‐1 endothelial cells was observed either in cell monolayer or cell suspension. The interaction in monolayer cells was more effective when compared with LipL41, a positive control protein previously studied [36]. The binding with monolayer Ea.hy926 and HMEC‐1 endothelial cells was dose‐dependent, reaching saturation at 200 nm with both cell lines. This suggests that the binding of rLIC_13355 to endothelial cells occurs mainly via ECM, but cellular receptors may also contribute.

Pathogenic *Leptospira*, like *Borrelia burgdorferi* and other pathogens, express proteins with multifunctional and redundant function, which is possibly part of their survival strategy. We present a putative lipoprotein, LIC_13355, previously annotated as hypothetical of unknown function, as an adhesin with the ability to bind to ECM and endothelial cells. Moreover, the increase in proteolytic power of *Leptospira* by PLA generation may contribute to overcoming barriers to tissue penetration through ECM degradation and immune system evasion by diminishing opsonophagocytosis. Altogether, our data support the notion that LIC_13355 is a putative leptospiral lipoprotein involved in pathogenesis.

## Conflict of interest

The authors declare no conflict of interest.

## Author contributions

IRMS, MBT, AFT, and ALTON conceived and designed the experiments. IRMS and MBT performed the experiments. IRMS, MBT, AFT, and ALTON analyzed the data. AFT and ALTON contributed with reagents/materials/analysis tools. IRMS, MBT, and ALTON wrote the paper. IRMS, MBT, AFT, and ALTON revised the paper.

## Data Availability

The data that support the findings of this study are available in Figs 1, 2, 3, 4, 5, 6, 7 and Table 1 of this article.
